# Conserved and Divergent Functions of the cAMP/PKA Signaling Pathway in *Candida albicans* and *Candida tropicalis*

**DOI:** 10.3390/jof4020068

**Published:** 2018-06-08

**Authors:** Chi-Jan Lin, Ying-Lien Chen

**Affiliations:** Department of Plant Pathology and Microbiology, National Taiwan University, 10617 Taipei, Taiwan; clin2@ntu.edu.tw

**Keywords:** protein kinase A, cAMP, *Candida albicans*, *C. tropicalis*, hyphal growth, white-opaque switching

## Abstract

Fungal species undergo many morphological transitions to adapt to changing environments, an important quality especially in fungal pathogens. For decades, *Candida albicans* has been one of the most prevalent human fungal pathogens, and recently, the prevalence of *Candida tropicalis* as a causative agent of candidiasis has increased. In *C. albicans,* the ability to switch between yeast and hyphal forms is thought to be a key virulence factor and is regulated by multiple signaling cascades—including the cyclic adenosine monophosphate/protein kinase A (cAMP/PKA), calcineurin, high-osmolarity glycerol (HOG), and mitogen-activated protein kinases (MAPK) signaling pathways—upon receiving environmental cues. The cAMP/PKA signaling pathway also triggers white-opaque switching in *C. albicans*. However, studies on *C. tropicalis* morphogenesis are limited. In this minireview, we discuss the regulation of the yeast-hypha transition, virulence, and white-opaque switching through the cAMP/PKA pathway in the closely related species *C. albicans* and *C. tropicalis*.

## 1. Introduction

*Candida* species are common causative agents of superficial and invasive fungal infections worldwide [1,2]. Of the more than 150 *Candida* species, *C. albicans* is the most prevalent and has been responsible for >60% of candidemia cases worldwide for decades [3]. Recently, the prevalence of non-*albicans Candida* species (NACs)—such as *C. tropicalis*, *C. glabrata*, and *C. parapsilosis*—in patients with candidiasis or candidemia has increased [4,5]. Among NACs, *C. tropicalis* is the first or second most commonly isolated species from patients with candidiasis, especially in tropical regions such as Brazil and Asia [6,7,8,9,10]. *C. tropicalis* is closely related to *C. albicans* based on genomic comparisons, but the natural habitats of these pathogens differ: *C. albicans* is a human commensal organism and is rarely found in the environment, whereas *C. tropicalis* is not only a commensal of the human oral cavity, but is also present in soil, compost, plants, beaches, and seawater in tropical or subtropical areas [11,12,13,14]. The ability of these species to occupy vastly different niches despite their genomic similarity may be due to distinct regulatory mechanisms upon receiving environmental cues, though knowledge of how this is accomplished is scant.

In order to propagate and cause disease, pathogens have developed several strategies to deal with the harsh conditions associated with survival within a host. In particular, morphological plasticity is an important tool for fungi to adapt to changing environments within a host. In *C. albicans*, the transition between yeast and filamentous (pseudohyphae and hyphae) forms as well as white-opaque switching have been observed and characterized. Cells that are locked in either yeast or hyphal forms have altered virulence, and white-opaque switching is associated with biofilm formation [15,16,17,18,19,20,21,22,23,24,25]. Environmental cues from different niches, such as the presence/absence of nutrients, serum, CO_2_, and high temperature, govern the morphological switch between different cell types in *C. albicans* [16,18,26,27,28,29,30,31,32,33]. These stimuli trigger various signaling transduction pathways, including the cyclic adenosine monophosphate/protein kinase A (cAMP/PKA), calcineurin, high-osmolarity glycerol (HOG), and mitogen-activated protein kinases (MAPK) pathways to promote or suppress morphological switching [15,16,34,35,36,37]. These morphological transitions also have been observed in *C. tropicalis* in various environmental conditions such as CO_2_, pH, and *N*-acetylglucosamine (GlcNAc) [38,39,40,41,42,43]. In this minireview, we focus on the involvement of the cAMP/PKA signaling pathway in the regulation of morphogenesis, virulence, and white-opaque switching in *C. albicans* and *C. tropicalis*.

## 2. cAMP/PKA Signaling Pathway

**Figure 1:**
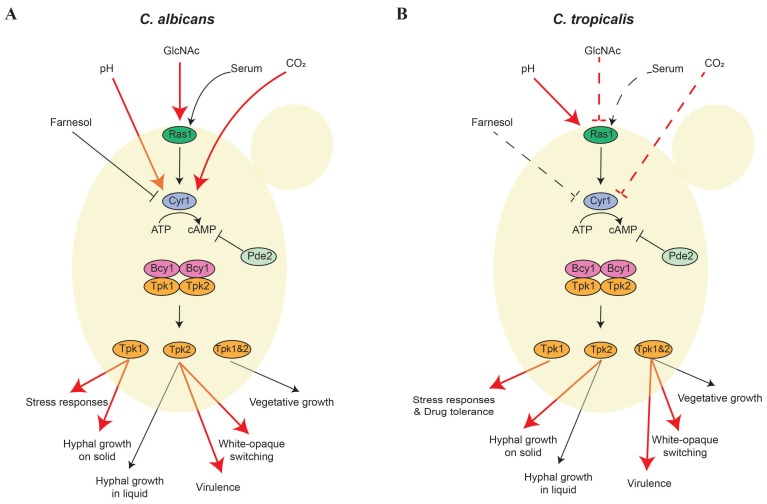
Regulatory models of the cyclic adenosine monophosphate/protein kinase A (cAMP/PKA) signaling pathway in *C. albicans* and *C. tropicalis*. Environmental cues activate cAMP/PKA in *C. albicans* (**A**) and *C. tropicalis* (**B**). Upon directly sensing environmental cues or being activated by Ras1, Cyr1 converts ATP to cAMP to bind the regulatory subunits of PKA (Bcy1) and liberate the catalytic subunits (Tpk1 and Tpk2). CaTpk1 is required for responding to stress (e.g., oxidative, osmotic, and thermal stressors), while CtTpk1 controls cell wall integrity and drug tolerance. In *C. albicans*, Tpk1 contributes to hyphal growth on solid media and Tpk2 is involved in hyphal growth in liquid media. In contrast, only Tpk2 contributes to hyphal growth on solid or in liquid media in *C. tropicalis*. In both species, Tpk1 and Tpk2 have redundant roles in vegetative growth. Furthermore, CtTpk1 and CtTpk2 share redundant functions in white-opaque switching and virulence, but in *C. albicans*, these two phenotypes are only controlled by Tpk2. GlcNAc induces both hyphal growth and white-opaque switching in *C. albicans*. In contrast, GlcNAc inhibits hyphal growth but promotes white-opaque switching in *C. tropicalis*. CO_2_ induces white-opaque switching in *C. albicans*, but inhibits the same transition in *C. tropicalis*. Environmental pH regulates hyphal growth via Cyr1 in *C. albicans*. In contrast, environmental pH controls white-opaque switching through Ras1-cAMP/PKA in *C. tropicalis*. Red lines and arrows indicate differences and dash lines represent these interactions have not been characterized between *C. albicans* and *C. tropicalis*.

The cAMP/PKA signaling pathway is conserved in eukaryotic organisms and has been well studied due to its crucial roles in pathogenesis and development in human and plant pathogenic fungi [44,45,46,47,48,49,50,51,52,53]. In *C. albicans*, the PKA holoenzyme contains two “inactivate” catalytic subunits (Tpk1 and Tpk2), each bound to a homodimer regulatory subunit (Bcy1) (Figure 1). Environmental cues such as glucose, amino acids, and serum can trigger the cAMP/PKA signaling pathway by activating adenylyl cyclase (Cyr1) to convert ATP to cAMP [44,47]. As a secondary messenger, cAMP binds to the regulatory subunits of PKA (Bcy1) and induces a conformational change that leads to dissociation and activation of the PKA catalytic subunits. These catalytic subunits then activate downstream targets such as Efg1 in *C. albicans* and *C. tropicalis* or Flo8 in *Saccharomyces cerevisiae* via phosphorylation to trigger various transcriptional regulatory circuits [29,41,45,54,55,56,57].

In *S. cerevisiae*, the cAMP/PKA signaling pathway is involved in the regulation of cell growth and cellular differentiation [58,59,60,61]. The *S. cerevisiae* PKA catalytic subunits (Tpk1, Tpk2, and Tpk3) show distinct roles in pseudohyphal growth. ScTpk2 positively regulates pseudohyphal growth upon nitrogen starvation, while the other two catalytic subunits, (ScTpk1 and ScTpk3) negatively influence pseudohyphal growth [62,63]. In *C. albicans*, the cAMP/PKA pathway plays critical roles in growth, morphogenesis, white-opaque switching, and virulence in murine models of systemic infection [16,18,64,65]. Upstream of PKA, adenylyl cyclase (Cyr1/Cdc35) is involved in filamentation and virulence, but this protein is not required for cell viability [28]. In contrast, in early studies, the regulatory subunit Bcy1 and the catalytic subunits Tpk1 and Tpk2 are essential for viability. The *bcy1/bcy1* mutant is only viable in a *tpk2/tpk2* mutant background, which has lower PKA activity compared with the wild type. Meanwhile, the conditional *tpk1/tpk1 tpk2/tpk2* mutant can be acquired, but has severe growth defects [66,67]. Recent studies have shown that the catalytic and regulatory subunits of PKA are not all required for cell viability [68,69]. By obtaining a *bcy1/bcy1* mutant that exhibited severe growth defects, Ding et al. showed that Bcy1 controls filamentation in *C. albicans* [68]. Soon after, Cao et al. obtained a *tpk1/tpk1 tpk2/tpk2* double deletion mutant that exhibited severe growth defects [69]—in line with previous studies in other pathogenic fungi such as *Cryptococcus neoformans* and *Aspergillus fumigatus*—showing that PKA is not essential for, but does regulate, fungal growth [49,70].

Although *C. tropicalis* is an emerging pathogen, studies related to its cAMP/PKA signaling pathway are limited. A pioneer study in *C. tropicalis* showed that Efg1, a transcription factor that is a potential downstream target of cAMP/PKA, is involved in the yeast-hypha transition, biofilm formation, and white-opaque switching [55]. Similarly, Efg1 also regulates morphogenesis and biofilm formation and is a downstream target of the cAMP/PKA pathway in *C. albicans* [18,56,57,71,72]. Hence, it appears that PKA has similar roles in *C. tropicalis* and *C. albicans*. The initial study of *C. tropicalis* PKA revealed that Tpk1 and Tpk2 have redundant functions in growth and filamentation, but the PKA catalytic null mutant was not viable. However, a few years later, two separate laboratories obtained *tpk1/tpk1 tpk2/tpk2* mutants that exhibited severe defects in cell growth, filamentation, and white-opaque switching [43,73]. In addition, Zhang et al. showed that loss of the adenylyl cyclase *CYR1* in *C. tropicalis* results in severe growth defects and reduced hyphal development [41]. Although the *C. tropicalis* adenylyl cyclase and PKA catalytic subunits have been identified and characterized, knowledge is limited on the functions of the regulatory subunit Bcy1 in growth and morphological transitions. Study of these PKA null mutants in *C. albicans* and *C. tropicalis* has facilitated the reevaluation of the global regulatory roles of the cAMP/PKA pathway.

### 2.1. cAMP/PKA Regulation of the Yeast-Hypha Transition and Virulence

cAMP is required to trigger the activation of the PKA pathway in response to various environmental stimuli, including GlcNAc, serum, amino acids, and CO_2_. Intracellular cAMP levels are tightly regulated by adenylyl cyclase (Cyr1) and phosphodiesterase (Pde2), which produce and degrade cAMP, respectively (Figure 1). In *C. albicans*, Cyr1 (which is not an essential gene) regulates hyphal growth induced by serum, CO_2_, or bacterial peptidoglycan [28,31,74], while the phosphodiesterase Pde2 plays a role in limiting intracellular cAMP levels. Loss of *PDE2* results in reduced hyphal growth and attenuated virulence in a murine model of systemic infection [75,76]. The *CaCYR1* and *CaPDE2* homologs are also involved in filamentation in *C. tropicalis*: The loss of *CtCYR1* and *CtPDE2* results in attenuated and enhanced filamentation, respectively [41]. Interestingly, Cyr1, but not Pde2, was demonstrated to impact cell growth (Table 1) [41].

In *C. albicans*, Tpk1 and Tpk2 have redundant roles in growth and dimorphic switching, but each has distinct roles in hyphal development in various filamentation-inducing conditions [67,72,77,78]. CaTpk1 contributes to filamentation on solid medium, while CaTpk2 is involved in hyphal growth in liquid medium [67,72,78,79]. In addition, the *tpk2/tpk2* mutant, but not the *tpk1/tpk1* mutant, exhibited reduced virulence in murine model of systemic infection [72]; interestingly, the *C. albicans tpk1/tpk1 tpk2/ptk2* double deletion mutant showed no hyphal growth in the various tested conditions, including solid and liquid Spider medium and Lee’s medium supplemented with glucose or GlcNAc as well as YPD with serum in liquid [69]. The virulence of the *tpk1/tpk1 tpk2/tpk2* mutant has not been characterized, but it is predicted to have attenuated or abolished virulence due to its severe defects in cell growth, hyphal growth, and white-opaque switching [69].

Similar to the *C. albicans tpk1/tpk1 tpk2/tpk2* double deletion mutant, the *bcy1/bcy1* mutant is not viable due to its high PKA activity in early studies. However, the *bcy1/bcy1* mutant was obtained in a *tpk2/tpk2* mutant background, which has reduced PKA activity, and showed defects in hyphal growth [66]. Recently, Ding et al. obtained a *bcy1/bcy1* null mutant in a *C. albicans* SN152 background that had two distinct phenotypes: smooth and filamentous [68]. Smooth *bcy1/bcy1* cells had defects in hyphal growth, while the filamentous *bcy1/bcy1* mutant exhibited hyphal growth indistinguishable from that of the wild type. Interestingly, the *byc1/bcy1* mutant was able to switch between these two phenotypes on YPD plates and in liquid SD medium, but had a preference for the filamentous phenotype under both conditions [68].

The first study of PKA in *C. tropicalis* was published in 2016 and suggested that Tpk1 and Tpk2 have redundant roles in filamentation and cell growth [41]. A year later, the same group reported severe defects in cell growth and white-opaque switching in the *tpk1/tpk1 tpk2/tpk2* double mutant, but did not characterize the impact on filamentation [43]. In 2018, Lin et al. demonstrated the viability of the *C. tropicalis tpk1/tpk1 tpk2/tpk2* mutant despite severe defects in cell growth, filamentation, and virulence in a murine model of systemic infection [73]. Surprisingly, the *C. tropicalis tpk1/tpk1* and *tpk2/tpk2* mutants showed virulence similar to the wild type in murine model of systemic infection. In addition to the redundant roles in cell growth and virulence, this group also found that Tpk1 and Tpk2 have distinct roles. For example, Tpk1 contributes to stress responses and antifungal drug tolerance, while Tpk2 plays a key role in filamentation, flocculation, and biofilm formation in *C. tropicalis* [73]. While CaTpk2 is required for virulence, CtTpk2 is not, suggesting divergent roles of Tpk2 in virulence between *C. albicans* and *C. tropicalis* (Table 1).

### 2.2. Regulation of White-Opaque Switching through the cAMP/PKA Pathway

In addition to transitioning between yeast and hyphal growth, *C. albicans* and *C. tropicalis* can undergo white-opaque switching—a heritable morphological change [40,43,80,81,82,83]. White cells (a, α, or a/α) form white, shiny, and smooth colonies on solid media. In inducing conditions such as GlcNAc or high CO_2_ concentrations, white cells transition to opaque cells and colonies that appear elongated, flat, and gray on solid media [17,84,85,86]. Similarly, *C. tropicalis* white cell colonies exhibit a rounded, smooth surface, while colonies of opaque cells have an elongated, pimpled surface [40,82]. However, slight differences have been noted between the opaque cells of *C. albicans* and *C. tropicalis*: *C. tropicalis* opaque cells cannot be stained with phloxine B whereas *C. albicans* opaque cells can, and *C. tropicalis* opaque cells are stable at high temperature (37 °C) whereas *C. albicans* opaque cells are not [82]. In addition to the differences noted above, high CO_2_ concentrations (5~20%) induce white-opaque switching and stabilize opaque cells at 37 °C in *C. albicans*, but inhibit the white-opaque transition in *C. tropicalis* [43,86,87].

Several studies have shown that the *C. albicans* cAMP/PKA pathway is highly involved in white-opaque switching [68,69,87,88]. The *tpk1/tpk1* or *tpk2/tpk2* mutant in *C. albicans* has a similar rate of white-opaque switching compared to the wild type in various culture conditions [69], but loss of *CYR1* results in low intracellular cAMP concentration, and a *tpk1/tpk1 tpk2/tpk2* mutant without obvious PKA activity showed severe defects in white-opaque switching [69,87,88]. In contrast, *pde2/pde2* knockout, *bcy1/bcy1* knockout, or *TPK2* overexpression results in cAMP/PKA pathway activation, thus promoting the white-opaque transition (Table 1) [68,69].

Similarly, the cAMP/PKA pathway plays an important role in white-opaque switching in *C. tropicalis*. Zheng et al. showed that loss of either *TPK1* or *TPK2* in *C. tropicalis* has no effect on white-opaque switching. However, activation of the cAMP/PKA pathway by *PDE2* deletion results in enhanced white-opaque switching, and deactivation of the cAMP/PKA pathway in a *cyr1/cyr1* mutant or PKA null mutant results in reduced or abolished white-opaque switching, respectively (Table 1) [43].

Although the cAMP/PKA pathway is involved in white-opaque switching in *C. albicans* and *C. tropicalis*, it is activated by different conditions in these close relatives. In *C. albicans*, cAMP levels are high (and thus promote white-opaque switching) in acidic or high CO_2_ conditions, whereas *C. tropicalis* cAMP levels are higher in alkaline and normal air conditions [43,69]. This difference might be due to the natural habitats of these fungal pathogens: *C. albicans* is a commensal that lives in different niches within humans with relatively high CO_2_ concentrations, while the environmental microorganism *C. tropicalis* is well adapted to normal air.

## 3. Conclusions

Although *C. albicans* and *C. tropicalis* are close relatives, their different natural habitats have shaped their regulatory systems via conserved signaling pathways (such as the cAMP/PKA pathway), which can facilitate the establishment of infection. In *C. albicans* and *C. tropicalis*, the cAMP/PKA singling pathway regulates the yeast-hypha transition, virulence, and white-opaque switching. In general, the components of the cAMP/PKA signaling pathway in the two species have similar roles in vegetative growth, hyphal development, and white-opaque switching. Interestingly, the catalytic subunit Tpk2 shows distinct roles in virulence between the species: CaTpk2, but not CtTpk2, is required for virulence in a murine model of systemic infection. Furthermore, different conditions induce or suppress the yeast-hypha transition and white-opaque switching in these two fungal pathogens, suggesting that cAMP/PKA might respond to different environmental cues. In addition to morphogenesis, the *C. albicans* cAMP/PKA pathway has been shown to regulate glycogen synthesis, energy metabolism, and mitochondrial activity [79,89]. Inhibition of mitochondrial activity resulted in reduced Ras1 and Cyr1 activity and consequently attenuated filamentation [89]. In addition, other proteins, such as Hsp90 and its co-activator Sgt1, regulate morphogenesis and drug tolerance in *C. albicans* [30,90], and small chemical compounds have also been found to control its morphogenesis via the cAMP/PKA pathway [91,92,93]. Furthermore, as the cAMP/PKA pathway is conserved in eukaryotic cells and regulates virulence in *C. albicans* and *C. tropicalis*, this pathway might be a promising target for antifungal drug development. A similar approach in the calcineurin pathway [94] has shown that calcineurin inhibitors exhibit antifungal activity alone or synergistically with other existing drugs, such as fluconazole, against *C. albicans* and *C. neoformans* [95,96]. Although the cAMP/PKA pathway is well characterized in *C. albicans*, knowledge of this important pathway in *C. tropicalis* is limited. Comparative studies on the roles of Hsp90 and small molecules in morphogenesis and virulence and the roles of cAMP/PKA in metabolic adaptation in *C. tropicalis* will contribute to the understanding of how these fungal pathogens utilize the cAMP/PKA pathway to cause human infections.

## Figures and Tables

**Table 1:** Conserved and divergent functions of the cAMP/PKA signaling pathway in *C. albicans* and *C. tropicalis*.

	Genotypes	*cyr1/cyr1*		*bcy1/bcy1*		*tpk1/tpk1*		*tpk2/tpk2*		*tpk1/tpk1 tpk2/tpk2*		*pde2/pde2*	
Phenotypes		Ca	Ct	Ca	Ct	Ca	Ct	Ca	Ct	Ca	Ct	Ca	Ct
Vegetative growth		A	A	A	-	WT	WT	WT	WT	A	A	A	WT
Hyphal development		A	A	E/A	-	WT	WT	WT/A	WT/E	A	A	A	E
White-opaque switch		A	A	E	-	WT	WT	A	WT	A	A	E	E
Virulence		A	-	-	-	WT	WT	A	WT	-	A	A	-

A, attenuated; Ca, *C. albicans*; Ct, *C. tropicalis*; E, enhanced; WT, similar to wild type; - not determined.
